# Counter-radicalization policies and policing in education: making a case for human security in Europe

**DOI:** 10.1016/j.heliyon.2020.e05721

**Published:** 2021-02-13

**Authors:** Gabriel O. Adebayo

**Affiliations:** Department of Education, Faculty of Educational Sciences, University of Helsinki, 00014 Helsinki, Finland

**Keywords:** Education, Counter-radicalization policy, European union, Extremism, Finland, Human security, Policing in education, Radicalization, Terrorism, United Kingdom

## Abstract

Research on counter-radicalization policies and policing in education in Europe is currently patchy and often focused on the United Kingdom. Scholars have observed that counter-radicalization policing in education is a threat to human freedom, human rights and dignity, and safe learning environments. However, scholars generally have not examined this issue from the viewpoint of human security. This paper examines the policing policy matter from the perspective of the personal security form of human security. The concern is that such a policing policy-related threat is antithetical to the concept of human security promoted by the United Nations (UN) and which the European Union (EU) and some European states had adopted. The study aims to find out how the current educational counter-radicalization initiatives and their effects could be used to argue for human security in Europe. The goal is to see how we can learn from past mistakes and improve future directions. The primary data are sourced from selected national, EU and UN policy documents, and a national media report. This work employs descriptive discourse analysis to analyse its data. The findings reveal that the present educational counter-radicalization policies of selected cases are grossly and/or explicitly deficient in the principles and language of human security. This has a negative impact on our understanding of the counter-radicalization policy effects in Europe. The study shows that the counter-radicalization strategy could trigger insecurity and negative security-oriented education for citizenship than we previously acknowledged in the literature. This piece suggests that the adverse consequences and tendencies could have been prevented had the appropriate human security elements been used in formulating and promoting the policy/strategy.

## 1 Introduction

### 1.1 Counter-radicalization, policing policies in education and human security

Countering radicalization through education is currently a major security policy of the United Kingdom (UK),^1^ Finland and the European Union (EU) among many countries and several intergovernmental organizations in Europe. It is part of their larger security strategy generally known as Preventing/Countering Violent Extremism (P/CVE) (Ragazzi, 2017). Their common aim is that education should help prevent young people from being drawn into violent extremism/radicalization and terrorism (Niemi et al., 2018), as some European states recently experienced terrorist attacks from their own citizens (Ragazzi, 2017; Thomas, 2016; Kundnani, 2012). Some European states’ counter-radicalization policies expect schools/teachers to police their students to prevent radicalization (Ragazzi, 2017). In this paper, policing is construed and defined as a P/CVE strategy, formulated and supported by any state or intergovernmental organization, asking or encouraging teachers/schools to be identifying radical students (i.e., students showing signs of radicalization) and referring them to security or non-security agencies for further P/CVE actions (cf. ibid.).

The EU (Council of the EU, 2009; Fakiolas and Tzifakis, 2019) and some of its Member States (e.g., Finland) have adopted the principles of human security promoted by the United Nations (UN) as part of their common security policy (Ministry for Foreign Affairs – MFA, 2009; cf. MFA, 1995). The UK explicitly integrated the principles of human security into its military curriculum and operations (Ministry of Defence, 2019). Nevertheless, an explicit use of human security language and principles in security-related policies/strategies (including the counter-radicalization issue) is generally lacking in Europe (cf. Kaldor et al., 2007; Fakiolas and Tzifakis, 2019). Human security, unlike human rights, has not enjoyed wide coherent and explicit currency in the EU security policy (cf. ibid.). A similar situation applies to Finland (Adebayo and Mansikka, 2018) as a Member State of the EU.

Moreover, the initiatives of the UN and its agencies to help prevent/counter violent radicalization through education do not explicitly promote human security. This is a concern in this paper, as “human security” was promulgated by a UN agency (United Nations Development Programme – UNDP) in 1994 (UNDP, 1994; Gasper and Gómez, 2015). Human security refers to the security of individuals and communities, expressed as freedom from fear, freedom from want (Kaldor et al., 2007, 273), and freedom from indignity (Gasper and Gómez, 2015, 102). Accordingly, human security could be defined as the protection of persons from threats or risks to their psychological or physical safety, dignity, and well-being (Tadjbakhsh and Chenoy, 2007; Adebayo and Mansikka, 2018).^2^

Generally, research on counter-radicalization policies in education from the perspective of human security is lacking in scholarship. While my previous research article (with Mansikka as secondary author) investigated policies relating to citizenship in Finnish religious education vis-à-vis human security, it does not adequately connect its findings to Finnish counter-radicalization policies (Adebayo and Mansikka, 2018). Besides, the previous article does not address the issue of policing in educational counter-radicalization policies. The previous study only briefly suggests that the current religious education curriculum has some value that appears as a counter-narrative and soft power (education) to prevent young people from being radicalized through religious fundamentalism and terrorism-oriented soft power (ibid.). Meanwhile, human security and counter-radicalization issues are not limited to religious education or Finland. Hence, this work examines some human security and counter-radicalization issues vis-à-vis education in general in selected national and intergovernmental cases in Europe. This paper inclusively addresses the issue of policing in counter-radicalization policies in education. Niemi et al. (2018) claim that the violent radicalization/extremism prevention programme in Finnish schools is not based on denying certain thoughts and opinions or judging the people presenting them (6). I suggest that this view is unbalanced, and I shall instead argue that Finland subtly promotes policing in its educational counter-radicalization policy.

Counter-radicalization policing in education/schools is now generating suspicions, fears and threats in certain quarters in Europe. Teachers are being seen as state informants targeting and working against Muslims (Dodd, 2015; Davies, 2014; Ragazzi, 2017; Faure-Walker, 2019). This is evident especially in the UK (ibid.), possibly because policing in education is statutory for schools/teachers in the UK, unlike in many other countries such as Finland (Ragazzi, 2017; Niemi et al., 2018). (Note that the UK is arguably the architect of the policing in educational counter-radicalization strategy in the EU [Ragazzi, 2017] and other parts of the Western world [Thomas, 2016].)^3^ Muslim children are now fearful of speaking freely in class, as they do not want to be identified/treated as potential Islamic terrorists (Dodd, 2015; Davies, 2016; Faure-Walker, 2019). Hence, there is a threat to their right to safe spaces in education (Ragazzi, 2017). Safe spaces in this context can be defined as education environments where students can freely learn, discuss and debate non-controversial and controversial issues of concern without fear (cf. CoE and EU, 2015).

Consequently, the policing strategy raises the need to query the notion of safe spaces/places in the educational counter-radicalization policy of some European states like the UK (HM Government, 2015) as to whether it is rhetoric or reality. The policing strategy is already threatening the dignity of some innocent students (children) as they are being suspected of being at risk of radicalism (cf. Ragazzi, 2017; Dodd, 2015). Essentially, the students'/parents’ freedoms from fear and indignity and right to psychological well-being are being threatened. The issues of freedom (cf. Kaldor et al., 2007) and psychological well-being at stake therein are intrinsically human security issues (cf. Gasper and Gómez, 2015).

Therefore, this research is interested in the psychological^4^ dimension of the personal security form of human security exemplified in freedom from fear (Adebayo and Mansikka, 2018; Lombardi and Wellman, 2012) and freedom from indignity (Gasper and Gómez, 2015). The paper aims to explore how the issues of fear and indignity resulting from the counter-radicalization initiatives in education in certain European quarters could be used to argue for human security. It focuses on the preventive aspect of counter-radicalization initiatives (Prevent) for basic education level (lower secondary school) students/pupils. Using cross-sectional design and descriptive discourse analysis, this research seeks to determine how human security lexicon can contribute to the discourse about anti-extremism in education in Europe and the world in general. The research aims to present findings that could help countries where the resultant fears and undignified experiences relating to the subject under investigation are currently visible (as seen in the UK) to amend their policy/strategy in favour of human security. The study also seeks to make some human security-driven analyses with a view to giving some guidance to European countries (like Finland) where fears resulting from counter-radicalization policy/strategy in education are not yet evident. This work attempts to show the need for a shift in the EU counter-radicalization policy/strategy in favour of human security language and principles for the benefit of all its Member States and Europe in general.

Given the above, this work employs a Finnish media report and selected counter-radicalization, educational and human security-bearing policy documents of the UK, Finland and the EU. Moreover, it uses some UN human security and P/CVE policy documents. The use of the UN policy is a necessity, as the UK (HM Government, 2009, 2018), Finland (MFA, 1995, 2009; Adebayo and Mansikka, 2018), and the EU (European Commission – EC^5^ ^2016^) recognize the UN as a strategic partner regarding security or counter-radicalization-related policies. Besides, the concept of human security employed in this work was, as noted above, promulgated by a UN agency - UNDP. The paper uses the example of the EU to demonstrate how intergovernmental organizations are impacting counter-radicalization strategies in European states. It uses all the selected cases to indicate how European countries and intergovernmental organizations have a mutual impact and similar pattern regarding the subject under investigation.

This study is significant, as policymakers and scholars have generally neglected the problem at the centre of this work. This deficiency seems to be negatively impacting our understanding of the educational counter-radicalization policy and its present and potential effects in today's Europe. While the principles of human security could be used in education to enhance security (Adebayo and Mansikka, 2018), this paper argues that the formulation and promotion of the current educational counter-radicalization policy of the EU, the UK and Finland (and in Europe by extension) are grossly and/or explicitly deficient in the principles and language of human security. The deficiency seems to have led to the policing in education that now appears to be antithetical to the principles and language of human security.

Moreover, this research gives a more balanced account of policing in Finnish education as against the one-sided view given by Niemi et al. (2018) (mentioned above). The inclusion of Finland in this work is significant, as up-to-date education research on radicalization and extremism in Finnish contexts is very scarce (Niemi et al., 2018). Incorporating Finland in this study becomes more necessary, as Niemi et al. (ibid.) do not analyse their findings about Finland in relation to human security. Including Finland is also apt as the country is seeking to promote a more in-depth approach to human security and find new ways of applying it (Adebayo and Mansikka, 2018, 467). Incorporating the UK herein seems to be appropriate too, as the country has also adopted human security. Incorporating the UK case in this study is necessary and strategic, as its counter-radicalization strategy informs and greatly influences that of the EU and Europe in general (Ragazzi, 2017; Thomas, 2016). Given that the research in this field is still patchy, and often focused on the UK (Ragazzi, 2017, 42) and that the focus on the UK itself generally lacks human security viewpoints, it seems the inclusion of the UK and Finland here is an added value to scholarship.

### 1.2 Research questions

The study seeks to answer the following specific research questions:

1. How do the preventive, counter-radicalization policing- and human security-related policy statements in the selected intergovernmental, national and media documents relate to, promote and/or negate human security in relation to the education of young people?
2. How could the notion of human security provide guidance in making necessary adjustments in the selected European countries where evidence of the resultant fear and indignity relating to counter-radicalization policing policy in education is currently visible or not visible?

### 1.3 Structure of the study

Given the above background, the next section presents the personal security form of human security in which freedom from fear and indignity finds its rightful expression as the conceptual framework of this work. The paper subsequently explicates radicalization as a flexible concept as well as the causes, origin, development, promotion and implications of counter-radicalization policing policy and strategy in the larger context of education in Europe where the cases of the EU, the UK and Finland play out. More specifically, this article briefly explicates how policing-embedded P/CVE strategy impacts the management of diversity in Europe and its implications for diversity and education. The explications are meant to reinforce the need to make a case for human security. It thereafter discusses the design, sources of data and method of analysis of this research. This is followed by the analysis of the results derived from the selected policy/media documents. The subsequent section presents a summary and further reflection on and discussion of the major findings of the work. The article concludes by stating the scope, limitations, and possible future directions of the research.

## 2 Conceptual framework

### 2.1 Freedom from fear and indignity: personal security in human security

The concept of human security came into the limelight through the 1994 UNDP *Human Development Report* (Tadjbakhsh and Chenoy, 2007); hence, it is generally credited to the UN and/or the UNDP. However, the concept does not necessarily represent the view of the UN/UNDP, as the 1994 UNDP report was based on the independent analysis of a team of experts led by Mahbub ul Haq, the Pakistani human development expert and theorist (Speth, 1994, iii–iv; cf. Streeten, 1995, xv–xvi).

Human security is now problematized (e.g., Tadjbakhsh and Chenoy, 2007; Gasper and Gómez, 2015) and has become a subject of research and policy interest (Kaldor et al., 2007; Seiple et al., 2013; Adebayo and Mansikka, 2018; Fakiolas and Tzifakis, 2019). Human security seems to attract so much interest because it challenges a once dominant security school of thought – the realist school of international relations (cf. Seiple et al., 2013, 2–3; Davies, 2014, 3–4). The realist school defined security in terms of traditional hard-power (troops and tanks) in which the major actors are the sovereign states (Seiple et al., 2013, 2). It sees military power as the primary means of securing the state. However, human security states that security is not restricted to the absence of threats to national territory and its institutions (Davies, 2014, 3–4). Human security advocates that security should be primarily about “human beings” and not the state (Davies, 2014; Kaldor et al., 2007).

For human security, traditional/state security is narrow, as it privileges sovereign states, state actors, their interests, and military solutions at the expense of individual citizens and their freedom and general well-being (Odutayo, 2016). In human security, the concept of freedom is pivotal in addressing every threat affecting people's security. Accordingly, the notion of freedom from fear and indignity is a cardinal value that human security adds to the security discourse (Gasper and Gómez, 2015; Gasper, 2005). This seems to be related to the fact that human security is “*the liberation of human beings from those intense, extensive, prolonged, and comprehensive threats to which their lives and freedom are vulnerable*” (UNDP, 2009, 23) (emphasis original). Unlike in state security, in human security the defence of human life is more important than the defence of land. Similarly, personal integrity is as important as territorial integrity in human security. Human security prefers peacekeeping to war-fighting, which is only seen as viable as a last resort (Dorn, n.d.). Hence, human security's broader view of security is complementary but not necessarily antithetical to state security (Tadjbakhsh and Chenoy, 2007).

Human security therefore poses new questions for the problem of security to which freedom from fear (ibid., 13–19; Adebayo and Mansikka, 2018) and freedom from indignity are an interconnected central answer (Gasper and Gómez, 2015, 102–103). “Security of whom?” “security from what?” (Tadjbakhsh and Chenoy, 2007, 13–17), and “security as perceived by whom?” are some of the new questions posed by human security (cf. Gasper and Gómez, 2015, 103; Gasper, 2005, 224–225).

I (with Mansikka) had previously noted that the question “security of whom?” (of human security) designates the individual rather than the state as the referent object of security. As such, the question “security of whom?” emphasizes personal security (Adebayo and Mansikka, 2018). Following Gasper and Gómez, personal security is one of the seven forms/categories of human security marking the beginning of the systematic account of human security as contained in the UNDP's *Human Development Report 1994*. The other six are: community, food, economic, environmental, health and political security. Meanwhile, the categorizations are not necessarily distinct, as each form/category of human security apparently has something to do with the security of persons (Gasper and Gómez, 2015). In human security, state security is deemed insufficient in an era when most violent conflicts are intrastate (not interstate) and, overwhelmingly, most casualties are civilians (Adebayo and Mansikka, 2018). Odutayo (2016, 373) puts it aptly as she notes that the essence of human security is to enable governments to see that people's security would generally enhance state security, thereby giving greater prominence to the issues of human rights and development.

As for the question “security from what?”, human security recognizes menaces beyond violence to include a host of threats. Security threats herein are inclusively associated with freedom from fear (Tadjbakhsh and Chenoy, 2007, 14–17; Adebayo and Mansikka, 2018) and freedom from humiliation and indignity (cf. Gasper, 2005, 225). Accordingly, the question security from what appears to address a key aspect of the psychological dimension of the personal security form of human security. This instance is particularly related to the rights of every person to freedom from fear, humiliation and indignity (cf. Gasper and Gómez, 2015, 102–103). As human security designates individual persons rather than the state as the referent object of security, the answer to the question “security as perceived by whom?” emphasizes the notion of security as perceived by individuals or a group of people rather than the state or interstate actors. This seems imperative, as human security suggests that a feeling of insecurity for most people today “arises more from worries about daily life than from the dread of a cataclysmic world event” (UNDP, 1994, 22). As such, the notion of “worries” herein reinforces the significance of the psychological perspective of the personal security category of human security.

Meanwhile, research and policy discourse often neglect psychological security in favour of the physical security aspect of the personal security form of human security, thereby downplaying the significance of the psychological dimension of human security (Gasper and Gómez, 2015). Nonetheless, the psychological aspects of “personal security” remain crucial elements in human security research and policy agendas (ibid., 103). Indeed, the human security discourse emerged in the 1990s as part of revisiting and rethinking the 1940s post-Second World War themes that emphasize the use of the psychological-related language of interrelated freedoms: “freedom from fear”, “freedom from want” and “human dignity” – hence also “freedom from indignity”. This development is meant to be beneficial to the post-Cold War era. Human security remains fundamental in lived experience, and is central to peace and the dignity of all persons. Psychological security is a prerequisite for adequately understanding personal security, as the “personal” is not restricted to the “physical” (Gasper and Gómez, 2015). People could feel insecure even when their physical persons are protected from physical threats. It is immoral to deny people physical safety or minimal material comforts; such denial will lead to instability. It is similarly immoral to deny people psychological security or human rights; such denial will also lead to insecurity (Lombardi and Wellman, 2012, 7).

Another question that human security poses for the problem of security is: “security by what means?” This question does not merely reject the primacy of the military (generally reactive) solutions promoted in state-centred security. It also holds that different instruments must be employed to address security menaces for short- and long-term effects (Tadjbakhsh and Chenoy, 2007, 18–19). Accordingly, the use of education in countering radicalization that could lead to terrorism seems to be human-security oriented, as human security is primarily proactive/preventive rather than reactive (Adebayo and Mansikka, 2018; cf. Ghosh et al., 2016). Possibly this led Davies to suggest that education and human security elements could help promote peace and security, avert crises and prevent violent radicalization/extremism (Davies, 2014).

### 2.2 Relevance of the conceptual framework

Explicit and wide use of the terms relating to human security is a necessity (Kaldor et al., 2007) in dealing with counter-radicalization issues. Kaldor, Martin and Selchow rightly pinpoint that concepts usually take hold only if they resonate, if they contain real meaning in providing a guide to action or a description of practice, and if action subsequently confirms the rightness of the description. As such, vague and empty linguistic vessels are usually unhelpful in seeking positive developments in policymaking, policy practice, policy analysis and policy research, as they usually obscure rather than clarify policy concerns. In a highly influential and strategic intergovernmental organization like the EU, an unambiguous use of concepts can help us have common understandings and expressions of issues (ibid., 273–274). Accordingly, this study is not satisfied with the common response – “[w]e already do human security, we just don't call it that” – of practitioners whenever discussions about the concept of human security comes up (ibid., 274). This article suggests that the use of the concepts of freedom from fear, of freedom from indignity and of personal security alongside their parent concept of human security in explicit terms can help clarify and take the discourse relating to counter-radicalization policies and issues in education in Europe a little further (cf. ibid.). This is more so, as the EU has adopted human security as part of its security policy (Fakiolas and Tzifakis, 2019; MFA, 2009). The above seems to be relevant for the EU (with its Member States), as the bloc seeks to improve its effectiveness and visibility as a collective global actor in the fight against terror (Kaldor et al., 2007, 273).

Similarly significant is that explicit usage of the terms relating to the psychological and other relevant intangible aspects of the personal security form of human security (mentioned above) would most likely complement human rights language, which is currently and explicitly entrenched in the discourse about counter-radicalization in education in Europe (e.g., Ragazzi, 2017). This seems tenable, as human rights and human security are mutually reinforcing in the pursuit of human dignity. Human security, by defining threats and duties, helps identify the rights at stake, while human rights identify the corresponding duties that promote human security (Tadjbakhsh and Chenoy, 2007, 123, 126; Adebayo and Mansikka, 2018, 463–464). Although human security, unlike human rights, cannot impose duties on others, it has an explanatory power that supports human rights issues (Tadjbakhsh and Chenoy, 2007, 127).

The question “security by what means?” that human security poses for security issues is useful in challenging the kinds of education counter-radicalization policies/strategies being used in the selected cases. One could thus ask: security by what type of educational means? This assists in querying the notion of policing in education. The scrutiny subsequently helps us to realize that the needed education is the one that has the potential to provide counter narratives (soft power) against violence-oriented radical ideologies – not policing education. It further helps show that such education should be presented to young people with a view to preventing them from being indoctrinated by and recruited into terrorist groups. The question also helps in reinforcing the fact that the needed education is one which can instil critical thinking, respect for diversity, and values for citizenship (cf. Ghosh et al., 2016; Macaluso, 2016).

The questions (security of whom? security from what? security as perceived by whom? and security by what means?) that human security poses for security are relevant in this paper. This is in the sense that they are helpful in analysing the policing policies in education with a view to making the people (primarily students in this case) rather than the state and/or intergovernmental actors the referent objects of security.

## 3 Radicalization, counter-radicalization and counter-terrorism policy and strategy

### 3.1 Radicalization: a flexible concept

The UK regards radicalization as the process by which a person comes to support or participate in terrorism or forms of extremism leading to terrorism (Taylor and Soni, 2017). The EU characterizes radicalization in a similar way (O'Donnell, 2016). Some scholars believe that such perspectives on radicalization are flawed and narrow, as radicalization is not necessarily a threat to security (e.g., Taylor and Soni, 2017, 241–242; ibid., 55). Associating radicalization with terrorism in the European governments' policy discourse only emerged in the 2000s. This is due to the failure of the use of force in fighting terrorism. The failure is reflected in the post-9/11 terrorist attacks in London, for example. It is believed among policymakers in Europe that such terrorist attacks occurred because the perpetrators were “radicalized” by terrorist ideology (Kundnani, 2012).

Meanwhile, radicalization could benefit human society (Organization for Security and Cooperation in Europe – OSCE, 2014; Macaluso, 2016, 2; Sukarieh and Tannock, 2015). For instance, those who championed the abolition of slavery were once regarded as radicals for opposing the prevailing worldviews of their societies (OSCE, 2014, 19, 35). Historically, radical ideas have propelled movements for workers’ rights in France and the UK; black activist movements in the United States; and the radical feminism and radical pacifism of the 1870s (Macaluso, 2016, 2). Mohammed Farouk decried the recent association of radicalization with violence/terrorism, as it was almost a rite of passage for students in Nigeria in the 1970s to become radicalized by taking on issues of social justice (British Council, 2015). For Sukarieh and Tannock, a significant goal of education is radicalization; hence the recent association of radicalization with terrorism is inappropriate (Sukarieh and Tannock, 2015).

Macaluso seems to have been right to suggest that the limited understanding of radicalization, particularly among policymakers, has so far led to ineffective/detrimental policies (Macaluso, 2016). Policing policy in education is a case in point here, as it now creates fears and indignity (antithetical to human security) among the students. Such limited understanding of radicalization seems to reinforce the need to examine P/CVE strategy from the human security viewpoint. Meanwhile, radicalization could be fairly defined as the process by which a person or group of persons comes to adopt increasingly “extreme” social, political or religious viewpoints and aspirations that challenge or undermine or reject the status quo; such extreme viewpoints and aspirations could be constructive or destructive (cf. Taylor and Soni, 2017; Macaluso, 2016; British Council, 2015).

### 3.2 Preventing citizens in Europe from supporting or participating in terrorism

The London terrorist attacks/bombings in 2005 (Thomas, 2016) up until the attacks in Paris in 2015 gave a significant place to domestic categorizations of terrorism in the political discourse in Europe. The discourse categorized such terrorism as “home-grown”. Home-grown terrorism is any terrorist threat coming from within the European societies themselves (Ragazzi, 2016, 724; Kundnani, 2012, 6), with the resulting need to prevent it (Kundnani, 2012; Ragazzi, 2017; Thomas, 2016; Qurashi, 2018).

Related to this, there is a focus on many European citizens travelling to the conflict zones in the Middle East to engage in conflicts. Following the 2015 attacks in Paris, there have been widespread concerns that individuals fighting with Islamist militants in the Middle East have become “radicalized” and could return to commit atrocities in Europe (cf. Mythen et al., 2017, 181; Mattsson et al., 2016, 256). The concerns seem understandable, as the four Islamic terrorists that perpetrated the 2005 London attacks/bombings were British citizens, brought up in England. These reinforce the domestic concerns and categorizations relating to terrorism (Thomas, 2016; Mythen et al., 2017). In bids to prevent future home-grown terrorism, many nations and intergovernmental organizations in Europe see education, rather than the military solution, as invaluable (cf. Ragazzi, 2017).

### 3.3 Europeanizing the UK's counter-radicalization policing in education: a call for human security

Counter-radicalization policing-oriented policy in education in Europe is generally influenced – directly or indirectly – by that of the UK (Ragazzi, 2017; Thomas, 2016). The policy expands the scope of counter-terrorism beyond the remit of traditional law enforcement agencies to non-security sectors such as education. In this instance, schools/teachers are expected to perform security-related functions (Ragazzi, 2017, 15–16; Davies, 2014, 149–150; Taylor and Soni, 2017, 242; Mattsson et al., 2016; Macaluso, 2016, 1). Meanwhile, the educational counter-radicalization policy in individual countries is not necessarily the same. The procedure used in each country depends on the nation's ethos, aims of education (Niemi et al., 2018, 3) and possibly most importantly the nation’s political and cultural worldviews.

Ragazzi noted that the EU Counter-terrorism Strategy of 2005 is virtually modelled after that of the UK. He noted also that the idea of policing-related strategy (Prevent) in education as a means of preventing radicalization, earlier conceived by the UK, is reinforced in the revised EU strategy for combating radicalization and recruitment to terrorism of 2014 (Ragazzi, 2017). Policing-related strategies are now part of the security-cum-education policy in Europe (ibid., 24–28, 52). The EU uses its Radicalization Awareness Network (RAN) to promote/support policing-related activities in education in the EU Member States (cf. ibid., 27) and non-EU Member States (EC, n.d.). Sometime in 2015 alone, over ninety educators from across Europe met at a conference on radicalization and education in Manchester where they “called” on schools in Europe to work together to prevent radicalization of students through a *Manifesto for Education* containing detective/policing elements (Sukarieh and Tannock, 2015). In their counter-radicalization policies, France (Niemi et al., 2018, 3; Ragazzi, 2017, 34, cf. 17) and Sweden (Mattsson, 2019) now ask their schools/teachers to police their students for possible signs of radicalization. Some other European countries (EU and non-EU), e.g., Belgium, Denmark, Finland, Slovakia, Spain, Switzerland, Kosovo,^6^ etc., have developed or are developing similar policies, although they are only now beginning to involve the education sector (Ragazzi, 2017, 17, 31–38).

Meanwhile, scholars have argued that the policy requiring schools/teachers to spot radicals, based on certain signs/indicators, would naturally make students entertain fears and hide their emotions and critical views. Hence, it constitutes a threat to their fundamental human rights, particularly their freedom of expression (Sukarieh and Tannock, 2015; O'Donnell, 2016; Mattsson et al., 2016; Thomas, 2016; Taylor and Soni, 2017; Ragazzi, 2017; Mattsson, 2019). The situation is made worse by the fact that some of the so-called signs/indicators (to spot radicals) are vague: “mundane behavioural changes in lifestyle and critical attitudes towards authorities and the values of mainstream society” (Ragazzi, 2017, 10, cf. 41–43; see also Taylor and Soni, 2017, 242). Some of the signs/indicators (e.g., students contesting the content of the teaching materials) (Ragazzi, 2017, 42) are antithetical to positive radicalization in education. For the academic community, the indicators/signs of radicalization used by the governments are based on contested scientific evidence (ibid., 21, 41). Educators fear that the governments' radicalization criteria could lead to unjustifiable referrals to the authorities and might be driving conversations underground (ibid., 41). Perhaps this is the reason why the EU with the Council of Europe (CoE) seeks to create “safe spaces” in the classroom where students can freely discuss issues of concern without fear (CoE and EU, 2015, 8).

Jagland seeks to:

Develop a “safe spaces” project around teaching controversial issues, with a view to drawing up guidelines for use in schools … that allow teachers and pupils to address difficult and controversial issues relating to faith, culture and foreign affairs, while respecting each other's rights and upholding freedom of expression. (Jagland, 2016, 12)

For him, the main purpose of safe spaces is perhaps to ensure that controversial opinions are not driven underground to develop – and possibly take root – away from the light of public scrutiny and open debate (Jagland, 2017, 5). Meanwhile, the idea of safe spaces/places is already in the educational counter-radicalization policies of some EU and/or CoE Member States, e.g., the UK (HM Government, 2015) and Finland (Finnish National Agency for Education – FNAE, 2018).

The above raises a question: how can violent extremist-oriented radicalization be prevented through security policies in education without infringing on the people's rights? (Ragazzi, 2017; Jagland, 2016). This article suggests that the language/principles of human security are invaluable here. The argument is that the strategy relating to the spotting of radicals in schools and unjustifiable referrals to the governments is not just a threat to the freedom of the victims from fear, humiliation and indignity, it is also deeply inimical to the psychological aspect of the personal security form of human security (cf. Gasper and Gómez, 2015).

### 3.4 More insights about the dominance of the UK's strategy in Europe

Dutch intelligence services were among the first in Europe to consider that terrorism should be addressed not only through law enforcement, but also through societal measures so as to address broader issues of polarization and integration between ethnic and religious groups in society. This is generally believed to have begun at the end of the 1990s (Ragazzi, 2017, 22). Following the London attacks/bombings of 2005, the UK became interested in the Dutch approach. Hence, from the mid-2000s onwards, the Netherlands and the UK became two prominent countries to promote preventive (“softer”) counter-terrorism both in Europe and internationally (ibid., 22–25, 31, 38, 43, 97). It is noteworthy that the 2005 attacks in London coincided with the EU British presidency. The coincidence gave the UK an opportunity to influence the bloc to prepare a European counter-terrorism strategy that was largely modelled on the one the UK had previously adopted (ibid., 25–26). Accordingly, the British rather than the Dutch counter-terrorism strategy became the dominant approach impacting the European counter-terrorism strategy (Ragazzi, 2017) and that of the West in general (Thomas, 2016; cf. Sukarieh and Tannock, 2015, 24).

The coordinating document of the UK counter-terrorism strategy (“CONTEST”) in this 21^st^ century was introduced in 2003 (Qurashi, 2018; cf. Ragazzi, 2017). CONTEST has four strategies/strands: Prevent, Pursue, Protect and Prepare (Ragazzi, 2017, 24; O'Donnell, 2016, 54–55). Prevent was meant to be the “hearts and minds” dimension of the overall CONTEST (Qurashi, 2018, 2). Prevent is meant to: (a) prevent people from supporting terrorism or being drawn into terrorism by ensuring that they are given appropriate support/advice (Ragazzi, 2017); (b) respond to the threat and ideological challenge of terrorism; and (c) work with sectors and institutions where there are risks of radicalization (Mythen et al., 2017, 183; Qurashi, 2018, 2).

While the UK's Prevent strategy significantly influences the policy of many other Western countries, it is highly controversial domestically (Thomas, 2016, 172). The Prevent strategy has always been the subject of considerably more criticism in the UK than the other strategies of the country's CONTEST, notably because of its policing elements in education (e.g., Qurashi, 2018). Hence, Mattsson's subtle position stating that Sweden has transferred the idea of counter-radicalization policing from abroad and from intergovernmental organizations into its education system without paying adequate attention to the local Swedish needs (Mattsson, 2019) seems to be narrow. Essentially, the notion of policing in education in countering the “supposed radicalization of students” is never the local need of any country. Indeed, policing-related Prevent programme/practice involving UK teachers or schools has been criticized and/or reported for its shortcomings (Kundnani, 2007, 20–21; Faure-Walker, 2019, 371–373, 375–379), including the creation of fear in the UK (Qurashi^7^ 2018, 10–12; Ragazzi, 2017, 51–52), the architect country of educational policing-embedded Prevent. Such a policing-related approach is herein categorized as antithetical to freedom from fear and indignity, a key component of human security.

### 3.5 P/CVE initiatives and policing in education versus diversity: eroding diversity?

How to manage diversity is part of the discourse about P/CVE in Europe. David Cameron (then Prime Minister of the UK), in his speech delivered at the 2011 Munich Security Conference in Germany, identified excessive tolerance of multiculturalism as a major cause of radicalization that led to terrorism in recent years. For him, Islamist extremism being held by perverted Muslims is the root cause of terrorist attacks in Europe (Cameron, 2011). He advocates “muscular liberalism” that must be “unambiguous and hard-nosed” to defend Western values (ibid.; Ragazzi, 2016, 724–725). Cameron's viewpoint on the “failure” of multiculturalism is like the one expressed by Germany's Chancellor Angela Merkel in 2010 (Ragazzi, 2016, 724–725; Chin, 2017). Merkel declared: “the multicultural concept is a failure, an absolute failure” (Chin, 2017, 237). For her, multiculturalism's blueprint had failed to establish clear guidelines to deal with immigrants whose practices contradict German liberal values (ibid., 285). Meanwhile, some Western (particularly European) authorities still support multiculturalism. For instance, Finland claims: it focuses on “strengthening multiculturalism, inclusion and equality” (Ministry of Education and Culture (MoEC) 2016, 4; cf. FNAE, 2018, 10).

It seems the anti-multiculturalist/assimilationist discourse in Cameron's speech is reminiscent of the UK's Prevent strategy. The current UK's Prevent policy emphasizes teaching of British values and policing people that have non-British values. The policy aim is to orientate those (regarded as “others”) who do not authentically (know how to) practise Britishness (Qurashi, 2018, 4). Teaching and policing for the purpose of promoting British values at the expense of diversity (of people, cultures, values, ideas and worldviews) is in fact mandatory for British teachers (James, 2019; Ragazzi, 2017). Teachers are thus finding it difficult to strike a balance between their professional training and the statutory tasks required of them (ibid.; Farrell and Lander, 2019). The recent counter-radicalization initiatives seem to have reignited “debates associated with assimilation, multiculturalism and integration” (James, 2019, 1–2). James notes that engagements with British values in schools should not impose what those in authority understand by being British. Such engagements should instead acknowledge and explore contestations of Britishness and what it means to be British for people of diverse cultures/ethnicities/religions. She notes further that this will enable schools to become critical sites for reflection, resistance and hopeful futures (James, 2019).

Chin describes the rejection of multiculturalism, declarations that multiculturalism has failed and promotion of uncompromising assimilation by some European policymaker(s) in fighting terrorism as “supremely unhelpful”. For Chin, such rejection/declaration/promotion is undemocratic, and curtails discussions of more inclusive conceptions of European society. She argues that there can be no such thing as cultural homogeneity in Europe; the challenge now is to think creatively about European diversity rather than settling for denial (Chin, 2017). Ragazzi rejects “muscular liberalism” and describes the impact of the recent counter-radicalization initiatives as “policed multiculturalism” resulting in labelling Muslims as a “suspect community”. For him, the issues of “muscular liberalism” and “suspect community” have negative consequences, as they promote assimilation rather than integration and remove fundamental questions about pluralism from political debate (Ragazzi, 2016).

While the British Prevent policy currently refers to other groups e.g., Irish and Northern Irish Republican paramilitary organizations and other forms of terrorism (O'Donnell, 2016) e.g., right-wing terrorism (Qurashi, 2018), many scholars believe that the thrust of the policy is about Islamic terrorism (e.g., ibid.). Hence, Prevent is believed to be targeting the “British Muslims” as those who are not authentically British and who must be policed and taught British values as a means of preventing radicalization leading to terrorism (ibid.; cf. Farrell and Lander, 2019; O'Donnell, 2016).

Framing Muslims as threats both “others” them and narrows down the public perception of Muslims so that they become identified with terrorist violence. Hence Islamophobia (Qurashi, 2018), assimilation, and erosion of diversity is normalized. This introduces the problem of the definition of extremism among European policymakers. The UK regards any vocal or active opposition to “fundamental British values” as extremism that must be fought. While the term “British values” includes democracy among other things, the list of values/behaviours that might be considered as extremism could be endless, hence making the notion of “British values” very vague (cf. Macaluso, 2016, 3). The Dutch Security Service similarly defines extremism as a growing willingness to pursue/support far-reaching changes in society that conflict with or threaten democratic norms (ibid.).

The problem with the view of extremism above in the context of educational counter-radicalization policing is that it gives policymakers, schools and teachers leeway to designate dissenting or diverse viewpoints as extremism. This is self-evidently an erosion of diversity and dissent. Cameron seems to have implicitly advised his fellow European (and other Western) leaders to do this: “Whether they [i.e., extremists] are violent in their means or not, we must make it impossible for the extremists to succeed” (Cameron, 2011). For Cameron, every extremist will engage in violence or terrorism if not contained early enough (ibid.). Sadly, the EU does not help the situation, as its law enforcement agency (Europol) leaves the definitions of terrorism-related matters such as terrorism and extremism to its Member States (Europol, 2018, 63).

Meanwhile, scholars have on many occasions found that the root cause of violence-oriented extremism/radicalization is rarely firm ideological commitment (notably to Islamist ideology). Rather, firm ideological commitment is usually found as its relational (secondary) cause in consequence of its root cause which may be hate speech, social exclusion, racism, perceived hostility of the Western governments towards Muslims around the world and excessive policing among others (Mythen et al., 2017; Kundnani, 2007, 2014). Hence, the idea that we must erode or deny diverse/dissenting values/viewpoints/ideologies through “muscular liberalism” and/or policing-oriented teaching to uproot violence-oriented radicalization/extremism seems to be misleading. Such a view could be counterproductive to any positive transformation agenda that education might bring (cf. Sukarieh and Tannock, 2015) in favour of human security.

## 4 Design and methods of study

### 4.1 Cross-sectional design

The choice of design for this study is cross-sectional. Following cross-sectional design principles (Bryman, 2004, 41–46; Ruane, 2016, 78–79; Labaree, 2019), this study:

• simultaneously collected qualitative data on the pertinent variables of the educational, policing, counter-radicalization and human security policies/strategies of the EU, the UK and Finland vis-à-vis qualitative data on human security and P/CVE in the UN's policies.
• simultaneously collected the data within a twelve-week^8^ period: 28.6.2019–25.09.2019.
• collected data on each variable without any follow-up with each document as per the data collected from it.
• examined the variables to understand the variation and patterns of association among the selected cases.
• provides a clear “snapshot” of the problem under investigation. (The idea of “snapshot” herein connotes a quick and brief analysis/discussion of the main variables of the subject matter. The notion of “snapshot” is not necessarily based on the duration of data collection but on the cross-sectional principle that disallows any follow-up on the data collected from each source.)

### 4.2 Sources of data, procedure and justification for selection

The sources of primary data in this paper are the policy documents of the UK, Finland, the EU and the UN and a Finnish Broadcasting Company (Yle) report. The documents were sourced by searching the institutional websites of the selected cases and references in previous studies. Purposive sampling procedure is used in selecting the documents, as the selection was based on some pre-specified (variable-based) inclusion criteria that could help address the article research questions (cf. Bryman, 2004, 333–334). The four tables (i.e., Tables 1, 2, 3 and 4) in this article contain the selected documents and justifications/criteria for their selection.

**Table 1:** UK's documents and justifications/criteria.

UK POLICY DOCUMENTS			
YEAR	AUTHOR	DOCUMENTS	JUSTIFICATIONS/CRITERIA
2004	Cabinet Office	A five-year CONTEST^9^	• Being the first UK coordinating CONTEST in the 21^st^ century (originally developed in 2003) • Being a guide impacting the formulation of subsequent counter-radicalization/counter-terrorism policy of the UK, the EU and many of its other Member States (including Finland)
2009	Home Affairs Committee	Report and update about CONTEST	• Containing relevant background regarding the pillars of the UK's CONTEST in the 21^st^ century • It recognizes “Prevent” (the main focus of this study) as the most significant pillar of CONTEST
2011	HM Government	*Prevent Strategy*	• The document deals with how the UK seeks to prevent radicalization • Containing definitions of extremism and radicalization that could threaten security-oriented education for citizenship and human security • It promotes policing in education
2015	Act of Parliament	Counter-Terrorism and Security Act, 2015	The Act stipulates policing as a statutory duty for school authorities in the UK
2015	HM Government	Revised Prevent duty guidance	This document maintains the definitions of extremism and radicalization earlier contained in the Prevent Strategy of 2011 and stipulates specific duties (e.g., policing and provision of safe spaces) for schools in England and Wales vis-à-vis the Counter-Terrorism and Security Act, 2015
2018	HM Government	Updated CONTEST	This CONTEST maintains Prevent, policing in education and the problematic UK view of extremism vis-à-vis education

9 This document was originally classified as “confidential”; it was later released to the public in response to a request made on the basis of the Freedom of Information Act. See: https://www.sacc.org.uk/press/2016/whitehall-releases-2003-counter-terrorism-strategy (accessed 5 September 2018).

**Table 2:** Finland's documents and justifications/criteria.

FINLAND POLICY DOCUMENTS			
YEAR	AUTHOR	DOCUMENTS	JUSTIFICATIONS/CRITERIA
2009	MFA	Finnish report on the human rights policy	Containing rare explicit Finnish views on human security
Amended up to 2010	Act of Parliament	Basic Education Act 628/1998	Being the subsisting Basic Education Act indicating that safe spaces in education are statutory in Finland
2012	Ministry of the Interior (MoI)	National action plan to prevent violent radicalization/extremism	• Being Finland's first action plan on how to prevent violent radicalization and extremism • Indicating Finland believes that radicalization/extremism could be positive • Containing one early view of Finland designating teachers as workers who could identify students with early signs of violent extremism
2016	MoEC	How to prevent hate speech and racism and foster social inclusion to prevent radicalization	Containing one statement indicating that Finland is supporting training of teachers on how to identify signs of radicalization
2016	Yle	Training teachers to identify radicalized youths	Indicating teachers as policing agents in education, as it reports that the Finnish police are training teachers on how to identify radicalized students
2018	FNAE	How to prevent violent radicalization in schools	• Being the first Finnish document solely committed to how schools can contribute to prevention of violent radicalization • Containing information that projects teachers as policing agents to identify signs of violent radicalization among students • Claims to promote safe spaces in education • Giving leeway to teachers as policing agents against radicalization • Containing inconsistency that could threaten safe spaces and human security
2018	MoI	National Counter-terrorism Strategy	• Being the third and subsisting Finnish counter-terrorism strategy • Containing an emergent Finnish concept of “Prevention”, a model of “Prevent” that is currently entrenched and elaborated in the UK's policies

**Table 3:** EU's documents and justifications/criteria.

EU POLICY DOCUMENTS			
YEAR	AUTHOR	DOCUMENTS	JUSTIFICATIONS/CRITERIA
2002	Council of the EU	EU framework decision on how to combat terrorism	• Being the first EU “Framework Decision” on how to combat terrorism in the 21^st^ century • It contains the “universal values” on which the EU is founded • Containing “universal values” that are relevant to the language of human security
2005	Council of the EU	EU counter-terrorism strategy	This document introduces the four pillars of the EU counter-terrorism strategy, including the “Prevent” pillar which is the main focus of this paper. The four pillars are similar to the ones originally prepared by the UK
2009	Council of the EU	EU security strategy	It contains two of the few instances where the EU incoherently and explicitly uses human security in its security policy
2014	Council of the EU	Revised EU strategy for combating radicalization and terrorism	• It recognizes “Prevent” as the essence of the whole effort to counter radicalization and terrorism • It recognizes and promotes teachers as policing agents to identify radical students for possible de-radicalization • It promotes training of teachers for policing
2014	EC	Communication to different arms and committees of the EU on prevention of radicalization and terrorism	It gives some policing-related perspectives of the EU on deradicalization
2017	European Parliament and the Council of the EU	EU directive on how to combat terrorism	• Being the subsisting framework on counter-radicalization policy of the EU • It now supersedes the 2002 “framework decision” • Containing the “universal values” on which the EU is founded • The “universal values” are relevant to the language of human security
2017	EU Committee of the Regions	Opinion of the EU about combat against radicalization and extremism	• It sees violent radicalization as a threat to the EU's perspective of universal values • Containing a definition of radicalization that could threaten security-oriented education for citizenship and human security • It presents the diversity of Europe as a major social and cultural asset rather than a security threat

**Table 4:** UN's documents and justifications/criteria.

UN POLICY DOCUMENTS			
YEAR	AUTHOR	DOCUMENTS	JUSTIFICATIONS/CRITERIA
1994	UNDP	Human development report	Being the promulgating document of human security by the UN
2003	Commission on Human Security	*Human Security Now*	It links human security with education
2009	UNDP	Human development report	Reinforces the significance of human security stated in the earlier UN documents
2017	United Nations Educational, Scientific and Cultural Organization (UNESCO)	Guide for policymakers concerning violent extremism	Promotes education as a means of preventing violent extremism, but generally discourages the kind of policing in education found in the strategies of the UK, the EU and Finland

### 4.3 Method of analysis

The method of analysis utilized in this work is descriptive discourse analysis. Descriptive discourse analysis is interested in describing how texts/talks are organized and how people pursue conversational goals and the strategies they use (Schreier, 2012, 46). Discourse analysis (descriptive or critical) deals with how language and social reality are interrelated (Schreier, 2012). Discourse analysts examine the language in texts with a view to tracing elements of discourses (Baker, 2006, 5; Bryman, 2004; Adebayo, 2019, 11). The assumption here is that language itself is not the reality. Language rather contributes to the construction of reality, particularly social reality (Schreier, 2012; Baker, 2006; Adebayo, 2019, 11). Hence, discourse analysts deal with how language is used and how it is not used (Schreier, 2012, 47; Adebayo, 2019, 11). Discourse analysis (descriptive or critical) is based on constructivist assumptions (ibid.; Bryman, 2004). Constructivist philosophy is built on the thesis of ontological relativity which holds that every tenable statement about existence depends on a worldview, and that no worldview about existence is absolute (Patton, 2002, 96–97).

The justification for descriptive discourse analysis in this study is as follows: Human security is constructivist-oriented (Tadjbakhsh and Chenoy, 2007, 87–89). Accordingly, the constructivist assumption that underlies discourse analysis enhances this article's ontological relativity view on security as seen in human security. As social phenomena, in constructivism, are in a state of constant revision (Bryman, 2004, 17), the constructivist assumption of descriptive discourse analysis is suitable for analysing how social actors (policymakers) are constructing counter-terrorism policies in response to the changing security challenges in today's Europe. The constructivist assumption in this discourse promotes a non-traditional security approach; this helps in analysing the EU (an intergovernmental organization), the UK, Finland and schools as collaborating security actors while individual students are treated as the referent objects of security (cf. Tadjbakhsh and Chenoy, 2007, 87–89). Moreover, the notion of human security language in this work helps in constructing and shaping reality (cf. Schreier, 2012).

The principles of descriptive discourse analysis are used in this article in the following ways: (1) The paper describes how the selected cases organized their counter-radicalization policing policy in education in relation to human security and how they pursue their goals (including the strategies they used). (2) This work traces and describes the language of human security in the selected documents as it contributes to the construction of social reality about counter-radicalization policing and related policies in education in the EU and its Member States. Accordingly, it analyses how the policy statements in the selected documents relate to, promote and/or negate human security in relation to the education of young people. (3) Related to this, the assumption of discourse analysis (in general) stating that language shapes reality is used to explicitly construct an alternative reality in favour of human security language as opposed to previous studies about counter-radicalization that have generally neglected this language. (4) This work employs the constructivist assumptions common to every form of discourse analysis to describe a glimpse of how the language of human security and counter-radicalization policies and policing in education is employed and how it is not employed (cf. ibid., 47) in the selected documents. Accordingly, the constructivist idea of the discourse is used to analyse how the human security notion could guide one to make necessary amendments to educational counter-radicalization policies having negative tendencies/consequences.

The excerpts selected for analysis were arrived at by tracing the discourse on the variables of this research aims and questions in the selected documents. Accordingly, human security, counter-radicalization policing in education and safe spaces were used as signposting variables. The searched words employed in tracing the discourse:

• on human security include freedom, freedom from fear, freedom from indignity, human dignity, universal and human security.
• on counter-radicalization policing in education include education, teachers, schools, students, pupils, police, signs of radicalization, radicalization, radical, extremism, refer, counter-radicalization, counter-terrorism and Prevent.
• on safe spaces include safety, safe, safe space and safe learning.
The discourse tracing reveals some patterns of association:
• between the UK, Finland and the EU, as none of them explicitly mentioned human security in their education counter-radicalization policies but only and scantily in their other security-related policies.
• between the UK, the EU and Finland, as the language relating to educational policing is present in their respective Prevent-related policies.
• between the UK and Finland, as the language relating to safe spaces in education contradicts that of policing in education in the counter-radicalization policies of both countries.
• between the UK and the EU, as they both unreservedly associate terrorism/violence with the definition of radicalization in their policies.

Also, the discourse tracing reveals some variations in the:

• peculiarity of the “British” in the “British values” mentioned in the UK policies versus the “universal” in the “universal values” mentioned in the EU policies.
• peculiarity of some positive perspectives in the Finnish policy on radicalization as against the unreserved association of terrorism/violence with the definition of radicalization in the UK's and the EU policies.
• peculiarity of the subsisting statutory position of the UK counter-radicalization policing in education as against that of others that is non-statutory.
• fact that human security and/or safe spaces in education seem to be more threatened in the UK policies than in Finland's and the EU policies.

The discourse on the patterns of association and variations is analysed vis-à-vis the UN promoted human security and a UN P/CVE guide.

## 5 Analysis of results

The analysis of the results in this research appears in three categorizations: (a) human security, universal values and their place in counter-radicalization policy, (b) preventive policy, strategy and policing in education: a threat to human security, and (c) safe spaces/places in educational counter-radicalization policy – rhetoric or reality? – a thoughtful question for human security. The categorizations are not exclusive; they are simply different viewpoints on the research focus, aims and questions stated above.

### 5.1 Human security, universal values and their place in counter-radicalization policy

The idea of combating/preventing terrorism in the counter-radicalization policy of the EU is founded on the need to uphold what the bloc calls “universal values”. The bloc reveals this in its first framework decision and the subsisting directive on combating terrorism in the 21^st^ century: “The European Union is founded on the universal values of human dignity, liberty, equality and solidarity, respect for human rights and fundamental freedoms. It is based on the … principles which are common to the Member States” (Council of the EU, 2002, L. 164/3; see also European Parliament and the Council of the EU, 2017, L. 88/6). The subsisting directive of the EU seems to be explicit about terrorism as an antithesis to the universal values: “Acts of terrorism constitute one of the most serious violations of the universal values of human dignity, freedom, equality and solidarity, and enjoyment of human rights and fundamental freedoms on which the Union is founded” (European Parliament and the Council of the EU, 2017, L. 88/6). Accordingly, terrorism is a threat to the basis of the EU's existence. Hence, radicalization leading to terrorism is by extension a serious issue in the EU and in Europe.

Each of the universal values (above) promoted by the Union seems to be essentially connected to human security, though the bloc is not explicit about this. Following the UN, “[h]uman security is a universal concern. It is relevant to people everywhere, in rich nations and poor [nations]” (UNDP, 1994, 22). Accordingly, this paper suggests that the values upheld as universal values (above) by the EU are incontrovertibly related to human security as the values are of universal concern.

Meanwhile, the universal values of primary concern in this work are fundamental freedoms, liberty and human dignity. To begin with, the fundamental components of human security usually find their expression in the term freedom. According to the UN, “[t]here have always been two major components of human security: freedom from fear and freedom from want” (ibid., 24). However, the freedoms in human security are not limited to these two components. This is perhaps the reason why some people believe that the main feature of the “global model” of human security “is that it guarantees human freedom [in general] within a framework of responsibility” anywhere in the world (UNDP, 2009, 29). Hence, we can logically add freedom from indignity (Gasper and Gómez, 2015). The UN conceptualization of human security vis-à-vis human dignity as far back as 1994 lends credence to this: “Human security is not a concern with weapons–it is a concern with human life and dignity” (UNDP, 1994, 22). Indeed, the UN emphasizes that “[h]uman security … reinforces human dignity” (Commission on Human Security, 2003, 4). Moreover, the conjunction of the issue of liberty with that of fundamental freedoms seems to correspond to a UN definition of human security stating that human security is “*the liberation of human beings from those intense, extensive, prolonged, and comprehensive threats to which their lives and freedom are vulnerable*” (UNDP, 2009, 23; Adebayo and Mansikka, 2018, 465) (emphasis original). Essentially, this reinforces the idea that liberty, fundamental freedoms and human dignity expressed in the universal values promoted by the EU is at the heart of the human security enjoined by the UN.

As an EU Member State, Finland, in 2009, noted the desire of the Union to use human security: “In December 2008, the EU adopted an updated security strategy stressing the importance of a broad security concept and adopting the principles of human security as part of its common foreign policy and security policy” (MFA, 2009, 37). However, this is yet to be widely manifested explicitly in formulating and promoting security policy (including education counter-radicalization policy) of the bloc. In fact, the Union's 2009 policy on security strategy only uses human security explicitly twice: “the EU already contributes to a more secure world. We have worked to build human security, by reducing poverty and inequality, promoting good governance and human rights, assisting development, and addressing the root causes of conflict and insecurity” (Council of the EU, 2009, 8). “We need to continue mainstreaming human rights issues in all activities in this field, including ESDP missions, through a people-based approach coherent with the concept of human security” (ibid., 22). These two instances clearly show that the explicit use of the concept of human security remains vague, as they do not reveal specific cases where and how the concept or its principles are or will be used. A mere explicit mentioning of human security vis-à-vis human rights, reduction of inequality and addressing of the root causes of insecurity in general without giving specific details does not suffice here. This observation reinforces the position of Kaldor et al. (2007) and Fakiolas and Tzifakis (2019) stating that explicit use of human security in the EU security policy is very scanty and incoherent.

The scanty explicit use of human security and its principles in the EU security-related policy seems to be modelled by the bloc Member States. Generally, the EU Member States’ security-related policies/strategies sparingly employ human security and its principles in explicit terms. Finland is a case in point here (cf. Adebayo and Mansikka, 2018), as the country claims that it is more interested in the practical application of the concept: “Finland seeks to ensure … practical applications of the *concept of human security* and reaffirming the *Responsibility to Protect*, particularly in the protection of civilian populations and conflict prevention” (MFA, 2009, 10–11) (emphasis original). While “*Finland*” claims that it “*will work to promote a more in-depth approach to human security and endeavour to find new ways of applying it in practice*” (ibid., 37) (emphasis original), the much sought-after “in-depth approach to human security” is yet to appear in explicit terms in its counter-radicalization policy/strategy in education and in general to date. As analysed below, the current practical application of human security by Finland in its education counter-radicalization policy designed to prevent conflict is potentially a threat to the security of the young people it is meant to protect. Broadly speaking, the strategy used in preventing terrorism through the EU-related counter-radicalization policy in education is a threat to liberty, fundamental freedoms and human dignity. Hence a threat to freedom from fear and indignity of the personal security form of human security of the students.

### 5.2 Preventive policy, strategy and policing in education: a threat to human security

#### 5.2.1 Prevent, prevention and policing policy in education

The lukewarm action about the use of human security and its principles in explicit terms seems to be impacting the formulation and promotion of Prevent in education. Prevent is one of the four pillars of the EU counter-terrorism strategy: “The four pillars of the EU's Counter-Terrorism Strategy … [are] prevent, protect, pursue and respond” (Council of the EU, 2005, 6). According to the EU, Prevent is meant “[t]o prevent people turning to terrorism by tackling the factors … which can lead to radicalisation and recruitment, in Europe and internationally” (ibid., 3). Nevertheless, the EU strategy/policy does not override that of individual countries:

the responsibility for combating radicalisation and recruitment to terrorism primarily lies with the Member States, this Strategy should help Member States develop, where relevant, their own programmes and policies, which take into account the specific needs, objectives and capabilities of each Member State. (Council of the EU, 2014, 4)

Prevent is the essence of the EU strategy for combating radicalization/terrorism. This is very evident in the 2014 revised version of the strategy: “The main objective of the strategy should be to prevent people from becoming radicalised, being radicalised and being recruited to terrorism and to prevent a new generation of terrorists from emerging” (ibid., 3).

The foregoing seems to reflect the UK CONTEST stating that the aim of Prevent is to “PREVENT the next generation of terrorists” (Cabinet Office, 2004); “Prevent … [aims] to stop people becoming terrorists or supporting violent extremism” (Home Affairs Committee, 2009, 8; see also HM Government, 2018, 8); “In some ways, the Prevent strand of CONTEST^10^ is the most important” (Home Affairs Committee, 2009, 10). Prevent is “[a] long-term but vital element in the strategy” (Cabinet Office, 2004). It is noteworthy that: “The Government [of the UK] developed its first comprehensive counter-terrorism strategy, known as CONTEST, in early 2003” (Home Affairs Committee, 2009, 4). Hence, the Prevent pillar of the EU counter-terrorism strategy incontrovertibly reflects the prior Prevent pillar of the UK counter-terrorism strategy.

Meanwhile, the UK-impacted EU Prevent pillar of counter-radicalization or counter-terrorism policy is now in turn impacting the counter-terrorism strategy of the EU Member States and European countries in general (e.g., Finland). In speaking of “Prevention” as one of the four “Strategic Policies”^11^ of its counter-terrorism strategy, Finland notes that “[t]he underlying causes, motivations and factors contributing to the proliferation of terrorism are prevented by identifying threats at an early stage, addressing risk factors and increasing awareness of the factors that contribute to the threat of terrorism” (MoI, 2018, 19). Finland explains further: “The focus in counter-terrorism is on prevention, which refers to addressing … other factors that may lead to … enlistment in terrorist groups” (ibid., 15). While Finland does not use “Prevent” verbatim as seen in the UK and the EU cases, its “Prevention” (the above-mentioned strategic policy) is clearly about stopping people from becoming terrorists, the hallmark of the UK and the EU Prevent respectively. The findings above seem to lend credence to the position of the previous studies stating that the counter-radicalization/counter-terrorism strategy in Europe is in many respects patterned after the UK's CONTEST (e.g., Ragazzi, 2017; Thomas, 2016).

The patterns of association between UK “Prevent”, EU “Prevent” and Finland “Prevention” include policing language/elements in education; this is particularly the case where the psychological aspect of the personal security form of the human security of the students comes under threat. To begin with, some policing language or element in education seems apparent in the following policy statements: The EU states that:

A wide range of sectors can help to prevent people supporting terrorism or promoting an extremist ideology linked terrorism or becoming terrorists. Training of teachers … [among other practitioners across different sectors] is a critical element of any successful programme to counter radicalisation. These practitioners … may be able to identify signs of radicalisation at an early stage, therefore they need to be aware of and understand signs of radicalisation to terrorism. (Council of the EU, 2014, 10)

The above indicates that the EU seeks to commit and train teachers as policing agents to counter radicalization. Teachers are thereby expected to identify signs of radicalization among their students early enough before the radicalization takes a violent form. In its bid to achieve and entrench this, the EU believes that:

We should encourage the development of awareness raising programmes and sector specific training modules for first line practitioners [teachers inclusive] to … help them offer support to individuals at risk or to refer them to specialised professionals for further help. (ibid.)

Considering the foregoing, the essence of training teachers as promoted by the Union is not just to identify early signs of radicalization among students but also to enable them to support individual students that might be at risk or to refer them to specialists-cum-professionals for further help. Note that being “at risk” herein suggests that individuals are believed to have been radicalized (i.e., have become potential terrorists).

The specialized professionals are expected to render help through what the EU calls “exit strategies” and “de-radicalisation”. According to the Union, exit strategies “can help radicals disengage (renounce violence without giving up the ideology underpinning it)” (EC, 2014, 7). However, to “de-radicalise [means to] … renounce both violence and the underlying ideology” (ibid.). “Exit strategies generally rely on individual mentoring consisting of psychological support and counselling” (ibid.). Given that exit strategies in education will normally come as a result of referrals from teachers, it suggests that the strategies can be collaborative between schools and other relevant sector(s): “The efforts to promote exit strategies may draw on cross sector collaboration between relevant authorities such as police, prison and probation services, social service providers, schools, etc.” (ibid.).

Reinforcing the findings of the previous studies (reviewed above), the problem with the policing elements of the Prevent policy is its negative tendencies and consequences: (1) creation of fears (Dodd, 2015; Davies, 2016; Ragazzi, 2017; Faure-Walker, 2019) and indignity among students, a problem that negates the principles and language of the psychological aspect of the personal security form of human security (UNDP, 1994, 2009; Commission on Human Security, 2003) and (2) creation of mistrust between citizens and state and between students and teachers (e.g., HM Government, 2011, 23, 31, 56), a problem that can be detrimental to education for citizenship and security. It seems the above-mentioned problems could be attributed to the lack of explicit use of appropriate principles and language of human security in formulating and promoting counter-radicalization policy in general.

The foregoing is very evident in the UK. Unlike in most European countries, the task of preventing people from being drawn into terrorism is currently a legal requirement for the school authorities in the UK: “A specified authority must, in the exercise of its functions, have due regard to the need to prevent people from being drawn into terrorism” (Counter-Terrorism and Security Act, 2015 26 § 1). “A specified authority is a person or body that is listed in Schedule 6” (ibid. 26 § 2). According to the UK government, “[t]he education and childcare specified authorities in Schedule 6 to the Act” are the proprietors of schools, pupil referral units and the registered early and later years childcare providers among others (HM Government, 2015, 11).

Specified authorities will need to demonstrate that they are protecting children and young people from being drawn into terrorism by having robust safeguarding policies in place to identify children at risk … Institutions will need to consider the level of risk to identify the most appropriate referral. (ibid.)

The UK Prevent seeks to “ensure that teachers and other school staff know what to do when they see signs that a child is at risk of radicalisation” (HM Government, 2011, 71; cf. HM Government, 2018, 36). “The Prevent duty requires education providers to have clear policies in place to safeguard students and build their resilience to radicalisation in schools” (HM Government, 2018, 36). More specifically for teachers, Prevent is meant to “establish a set of standards for teachers which clarifies obligations regarding extremism” (HM Government, 2011, 71). Hence, it is apparent that the statutory responsibility of the school authorities in the UK is so strong that each school is expected to draw up its own micro-policy of Prevent with a view to spotting early stage radical or extremist behaviour among the students.

However, the UK counter-radicalization policing policy designed for “protecting children and young people” is now a source of worry for some people: “There have been allegations that previous Prevent programmes have been used to spy on communities [particularly the Muslim community]” (ibid., 23). In fact, the parents of Muslim students who had been spotted (under the UK Prevent programmes) in schools as exhibiting alleged early radical behaviour and traits by their teachers have claimed that their children are being presumed guilty of terrorism due to their Muslim heritage. This indicates a case of mistrust between citizens and the state and students and teachers (Dodd, 2015; Ragazzi, 2017). The British government itself acknowledges the trust deficit: “Trust in Prevent must be improved” (HM Government, 2011, 23). Meanwhile, the UK claims that: “We can find no evidence to support these claims.” It also notes that “Prevent must not be used as a means for covert spying on people or communities” (ibid.). However, the UK Prevent at the outset singles out only the Muslim youth in the UK as those to prevent from being radicalized: “BUT WE WILL DO MORE TO: Prevent the radicalisation of Muslim youth in the UK” (Cabinet Office, 2004; emphasis original). This suggests that the government is apparently biased against Muslims, at least at the beginning of Prevent. Hence, the fears and concerns of Muslim students and their parents and the mistrust exemplified between the parties mentioned above is understandable and not necessarily misplaced. The development seems to reinforce the belief in certain quarters in the UK that the main aim of Prevent is to project the “British Muslims” as the potential violent radicals needing de-radicalization (Qurashi, 2018; Farrell and Lander, 2019; O'Donnell, 2016; Kundnani, 2007).

#### 5.2.2 Human security threatened in educational terrorism preventive strategy

The major issue here is that the policymakers did not take the language of human security into consideration in conceiving Prevent. This seems to be harmful to the students and their parents, as it limits their personal security to the physical at the expense of the psychological, places their freedom from fear under threat and places the dignity of innocent students in a bad light. The claim of the UK – “Taking early action to protect people from radicalisation is not the same as surveillance or intelligence gathering. It is intended to pre-empt not to facilitate law enforcement action” (HM Government, 2011, 56) – does not obliterate the problems of fear of being spied on and the indignity of being identified as a radical. This is true in the sense that this aspect of Prevent (rightly and/or wrongly) identifies specific students as potentially dangerous and real “radicals” needing “de-radicalisation” to attain cognitive and/or behavioural change:

This area of *Prevent* is based on the premise that people being drawn into radicalisation and recruitment can be identified and then provided with support. The purpose of that support is to dissuade them from engaging in and supporting terrorist-related activity. This support is sometimes described as ‘de-radicalisation’, a term which is sometimes used to refer to cognitive or behavioural change. (ibid.)

Hence, this discourse suggests that policymakers/practitioners need to employ the language of human security wherein students' and/or parents’ freedoms from fear and indignity can rightfully find their expression regarding the counter-radicalization issues. This article holds that the new questions that human security poses for the problem of security are indispensable in addressing these issues.

The task of identifying and referring radical students for de-radicalization is not yet a statutory responsibility for Finnish teachers or school authorities. Finland, nevertheless, subtly engages its teachers in policing in education, though the government seems unwilling to admit this. For instance, Finland states that it does not want teachers to label or identify students with regard to radicalization:

It is very important that no young person is labelled and that no misunderstandings take place and no overinterpretations are made. We do not wish to send teachers in Finland a message that schools should especially be looking for or identifying young people who are becoming radicalised. (FNAE, 2018, 13)

While the foregoing suggests that Finland expressly rejects policing in education, the country also mentions teachers as part of those that could identify early signs of extremism: “In their basic work, teachers … [among other professionals] face situations in which it is possible to identify early signs of violent extremist thinking” (MoI, 2012, 20). Moreover, Finland is subtly using its police authorities to train schoolteachers and educators to spot students showing signs of radicalism and extremism:

Finnish police will begin training schoolteachers to better identify young students who show signs of extremism and radicalisation. … Police will begin training education sector employees more comprehensively nationwide beginning next year. … The practical training is a pre-emptive police measure overseen by security professionals. The training is already underway in parts of the country. (Yle, 2016)

The cited Finnish counter-radicalization approach suggests that teachers and educators in Finland are not only engaged in policing in education, they are also doing it under the guidance of the state police and other security professionals. In what appears as if Finland is pre-empting how its citizens may react to such training, it says: “The police training is voluntary” (ibid.).

It seems that the idea of training teachers or educators on how to identify radical students is reminiscent of the above stated EU policy (Council of the EU, 2014). Such training is part of the UK Prevent. For instance, the Scottish police gave similar training to education staff in Scotland (HM Government, 2011, 58).

In another subtle attempt, Finland notes:

There is no exhaustive list of distinctive signs of violent radicalisation. Teachers and people who work with children and young people must know how to spot changes that give cause for concern in children's and young people's behaviour and must always refer the matter to student welfare services. (FNAE, 2018, 7)

Although the language of the foregoing does not explicitly call for de-radicalization of young people, it is nevertheless implied. One could inquire: what would the student welfare services do with the students who might have been earmarked as potentially dangerous radicals? The answer is:

In schools, … the pupil welfare group … assesses the situation and contacts the police if necessary. The police ensure that, if it is not possible to or there is no reason to take any police measures, the person is directed to the services provided by the local authority or organisations. (ibid., 17)

A similar approach is also found in the Finnish MoEC policy statement: “Teaching staff and other professionals are trained to spot signals related to … radicalisation and how to address these issues” (MoEC, 2016, 5). Given the above, it seems Finland is not revealing enough about how teachers, schools and the government are collaboratively addressing the issues of students that may be identified as radicals or potential radicals. Meanwhile, the Finnish ideas of changes in behaviour of children that could call for concern or that might be categorized as signs of violent radicalization can also be difficult to pin down. As seen in the UK case, these kinds of policy seem to give leeway to the government and teachers to determine which children and young people are supposedly violent extremists and radicals (cf. Ragazzi, 2017; Taylor and Soni, 2017). All these appear to be a threat to the dignity and psychological aspect of the security of innocent students and an antithesis of safe spaces in education^12^ (cf. Ragazzi, 2017; Jagland, 2016).

The findings above confirm that there are patterns of association between the EU, the UK and Finland regarding education counter-radicalization policing policies. The findings also confirm my viewpoint stated at the outset of this article, as they indicate that the position of Niemi and her colleagues stating that Finland's (unlike many countries') terrorism prevention programme in schools does not seek to deny certain thoughts and opinions or judge the people presenting them (Niemi et al., 2018) is misleading. While the issues of fear, assaults on the dignity of persons and mistrust seen as the resultant outcomes of the UK Prevent are not yet seen in connection with Finnish “Prevention”, Finland seems to be somewhat on the path of the UK and the EU counterproductive counter-terrorism strategy. This means that Finnish counter-radicalization policy also lacks appropriate language and principles of human security in its formulation and promotion. Accordingly, Finland may not escape what befell the UK if it continues with the present trend. This seems to be significant for Finland, as it has desired “*a more in-depth approach to human security and … new ways of applying it in practice*” since 2009 (MFA, 2009, 37) (emphasis original).

Central to this paper is that the role of education is not to police students; policing in education is not the right approach to enhancing citizenship, safety and human security. Following UNESCO:

The role of education is … not to intercept violent extremists or identify individuals who may potentially become violent extremists, but to create the conditions that build the defences, within learners, against violent extremism and strengthen their commitment to non-violence and peace. (UNESCO, 2017, 22)

Hence, the relevant national and intergovernmental stakeholders need to duly imbibe human security and UNESCO's viewpoint (above) in educational counter-radicalization matters. Otherwise, there could be unsafe spaces/places and related transgressions against young learners across the schools in the European contexts under investigation.

### 5.3 Safe spaces/places in educational counter-radicalization policy – rhetoric or reality? – a thoughtful question for human security

The UK's guidance on Prevent claims: “Schools should be safe spaces in which children and young people can understand and discuss sensitive topics” (HM Government, 2015, 11). According to the UK, such sensitive topics include “terrorism and the extremist ideas that are part of terrorist ideology, and learn[ing] how to challenge these ideas. The Prevent duty is not intended to limit discussion of these issues” (ibid.).

Finland uses the term safe places to convey something similar to that of safe spaces seen in the UK Prevent, hence indicating a pattern of association. In its counter-radicalization policy, “Finland emphasises schools’ fundamental task to provide children and young people with a safe place for high-quality learning” (FNAE, 2018, 11). Finland expects controversial issues to be open for discussion without jeopardizing safe spaces/places for the students:

It is important to make young people feel safe and accepted in the school environment and allow them to express their thoughts without adults feeling uncertain or being provoked by it. Interaction must be open and non-judgemental and it must be based on honesty and trust. (ibid., 13)

Provision of safe places for students/pupils is a subsisting statutory responsibility for education providers in Finland: “A pupil participating in education shall be entitled to a safe learning environment. The education provider shall draw up a plan … for safeguarding pupils against … harassment, execute the plan and supervise adherence to it and its implementation” (*Basic Education Act 628/1998: Amendments up to 1136/2010* 29 § 1 & 2). Hence, the idea of safe places in the counter-radicalization policy in Finnish education could be described as a reflection of the Finnish Basic Education Act.

However, the policing in counter-radicalization policy in education seems to project the idea of safe spaces/places in schools as rhetoric rather than reality. The countries' counter-radicalization strategies are descriptively inconsistent with their notion of safe spaces/places. For instance, the UK Prevent requires broad and balanced views of teaching school curricular subjects: “All schools are required by law to teach a broad and balanced curriculum which promotes the spiritual, moral and cultural development of pupils and prepares them for the opportunities, responsibilities and experiences of life” (HM Government, 2011, 65; see also HM Government, 2015, 10). In the same breath, the same UK Prevent does not welcome dissenting views to what it calls “fundamental British values”. It regards such dissenting views as extremism. “Extremism is vocal or active opposition to fundamental British values, including [i.e., not limited to] democracy, the rule of law, individual liberty and mutual respect and tolerance of different faiths and beliefs” (HM Government, 2011, 107; see also HM Government, 2015, 2, 21 for the same UK's definition of extremism). The UK authorities “note that previous *Prevent* documents used the phrase ‘violent extremism’. The review found that the term is ambiguous and has caused some confusion in the past” (HM Government, 2011, 25). Hence, the UK Prevent now says: “‘Non-violent extremism’ is extremism, as defined above, which is not accompanied by violence” (HM Government, 2015, 21). “The Government is clear that there is no place for extremists [violent or non-violent] in any school” (HM Government, 2011, 70). The British government reiterates this in the 2018 version of its CONTEST: “We protect the values of our society … by tackling extremism in all its forms” (HM Government, 2018, 23). The societal values herein are maintained as “fundamental, pluralistic British values” (ibid., 78).

An implication of this inconsistency is that teachers could report any student that may express opposing views to the “fundamental British values” as extremists or radicals needing de-radicalization. As suggested above, designating students as extremists in such a case would be a matter of teachers' or schools’ dispositions/discretion, as “fundamental British values” could be endless and controversial.

The idea that vocal opposition to “fundamental British values” is extremism that must not be tolerated also contradicts basic education as a means of realizing students’ human security. This concurs with the 9^th^ recommendation of the UN Commission on Human Security on how to advance human security: “Basic education and literacy are vital … for empowering students, keeping them safe and giving them a broader world view” (Commission on Human Security, 2003, 140). The Commission further advises:

Curricula should cultivate respect for other races, faiths, cultures and viewpoints … They should also teach students to reason, to consider ethical claims and to understand and work with such fundamental ideas as human rights, human diversity and interdependence. … [S]tates that champion human security should check that their own curricula cultivate mutual respect and emphasize the multiplicity of identities that people hold. Particular care should be given to eradicating inflammatory messages … (ibid., 141)

The UK's viewpoint on extremism, disallowing vocal opposition to “fundamental British values” and promoting democracy, appears to be self-conflicting, as democracy permits vocal (non-violent) opposition, even against “good” values. Fundamental “British values” appear to be varied from “universal values”, as “defining what is ‘British’ about such values is highly problematic, even before Britain's controversial past and present world role is considered” (Thomas, 2016, 184). More specifically, “British” is not a generally descriptive name for the human race. Essentially, the UK's policy designed to protect “fundamental British values” seems to lack sensitive, respectful and tolerant language for inherent diversity of the human race; this diversity entails nations, cultures, races and diverse viewpoints. The development appears to promote anti-multiculturalist/assimilationist discourse and erosion of diversity that one may deduce from Cameron's doctrine of muscular liberalism (Cameron, 2011). This could be divisive, inflammatory and counterproductive to security in a diverse country like the UK.

Unlike the UK, the EU does not restrict its acceptable values to those of any specific grouping of humans in construing extremism or radicalization. For the EU, “violent radicalisation … presents a threat to [all] citizens in Europe as well as to Europe's universal values” (EU Committee of the Regions, 2017, C 17/33). Moreover, the EU believes that “the diversity of Europe is an essential element of its social structure and a key cultural asset” (ibid., C 17/35). As such, the EU enjoins: “We must, inter alia, focus on … promoting inter-cultural dialogue, strengthening education to enable opportunities and critical thinking, and promoting tolerance and mutual respect” (Council of the EU, 2014, 6). Juxtaposing this EU statement with the UK's views on extremism, the UK seems to suggest that critical thinking means destructive extremism/radicalization, as critical thinking will naturally involve thinking beyond British values. British policymakers seem to be narrowing the students' values domain of thinking to Britishness rather than humanness. The development appears as if British values possess the monopoly of good civic matters. Accordingly, the current UK policy negates the language and principle of human security that could promote the students' freedom from fear to engage in critical thinking. This point is very significant, as the education (soft power) needed in providing a counter narrative to violent extremism/radicalization is the one that can instil critical thinking, respect for diversity, and appreciation of broad values of citizenship (cf. Ghosh et al., 2016; Macaluso, 2016). The UK development suggests that safe spaces for the students are only guaranteed for as long as they give no dissenting opinions against fundamental British values. It appears, in this sense, that the notion of safe spaces in education is merely rhetoric.

The UK's current definitions of extremism (above) and radicalization (below) seem to be antithetical to safe spaces in education. The UK unreservedly associates radicalization with terrorism: “*Radicalisation* refers to the process by which a person comes to support terrorism and forms of extremism leading to terrorism” (HM Government, 2011, 108; see also HM Government 2015, 21) (emphasis original). Given the history of this research, the definition seems to negate the original viewpoint on radicalization that emphasizes social justice (Taylor and Soni, 2017; O'Donnell, 2016; Macaluso, 2016; Sukarieh and Tannock, 2015; OSCE, 2014). It appears the UK could learn something from some other countries (e.g., Finland) in this respect, as Finland does not declare opposing views to fundamental Finnish values as extremism. In fact, Finland still believes that radicalization and extremism could benefit humanity because:

Radical ideas in themselves do not constitute extremism. Radicalism can be a positive, developmental and socially progressive force. … Non-violent extremism that refrains from violence is not, in itself, objectionable, although in terms of social cohesion extreme thinking arising from intolerance and hate is a cause for concern. (MoI, 2012, 9)

There is a somewhat similar pattern of association between the UK and the EU regarding the definition of radicalization, as the EU associates it with violence: The EU defines:

‘radicalisation’ as a phenomenon of people who regard the use of violence as legitimate and/or use violence themselves in order to achieve their political objectives which undermine the democratic legal order and the fundamental rights on which it is based. (EU Committee of the Regions, 2017, C 17/34)

Essentially, the EU too could learn something positive from Finland in defining/construing radicalization. All these indicate that Finland's perspective on radicalization and extremism seems to be more balanced than that of the UK and the EU, though its general counter-radicalization strategy in education is also not perfect. It also suggests that Finland's perspective on radicalization and extremism somewhat aligns with the scholarly position that disagrees with the recent association of radicalization with terrorism (e.g., Taylor and Soni, 2017; O'Donnell, 2016; Macaluso, 2016; Sukarieh and Tannock, 2015). Hence, the UK and the EU need to consider and accept the fact that radicalization and/or extremism are/is not originally about violence and not necessarily about violence in today's world (cf. Taylor and Soni, 2017; O'Donnell, 2016; Macaluso 2016; OSCE, 2014).

It is not good enough that the UK “recognise[s] that programmes comparable to Prevent are being run in other countries under the banner of preventing or countering violent extremism” (HM Government, 2011, 25). The position of this paper is that radicalization and extremism are essential to human civilization, but they can become violent when they are abused.

Meanwhile, Finland itself is not giving enough room for safe places due to its subtle policing in countering radicalization. Be it voluntary or statutory, the Finnish idea of using the police to train teachers/educators on how to spot radical students cannot enhance safe spaces. The Finnish policy wherein schools/teachers are given leeway to spot changes and behaviour of the students that may call for concern cannot be described as pro-safe places, as it could be psychologically threatening to young people and lead to assaults on the dignity of the students. This indicates that the Finnish policy on safe places in education also looks like rhetoric rather than reality.

We cannot rely on Jagland's (2016) safe spaces project in addressing the problems associated with policing in education, as safe spaces and safe place are already explicitly mentioned in the educational counter-radicalization policies of the states under investigation. This makes Jagland's safe spaces suggestion looks like more rhetoric. Safe spaces/places in education seem to have been viewed from the perspective of the states and intergovernmental actors rather than the students/parents. This is apparently a human security problem. Hence, it is also necessary to address the issue of safe spaces/places with the instrumentality of the new questions that human security poses for the problem of security.

## 6 Summary, further reflection and discussion

This paper shows that the scanty use of human security in explicit terms in the EU's, the UK's and Finland's security-related policy could not adequately promote human security in the education of young people. Central to this is that human security is not mentioned explicitly in any specific education counter-radicalization policy of the EU, the UK or Finland. This deficiency has been suggested to have possibly brought about the fear- and indignity-inclined policing issue in formulating and promoting the education counter-radicalization policies/strategies of the three cases. The findings indicate that the policing policy negates the human security of the students. This study finds the policing policy/strategy contradictory to the notion of safe spaces/places in the educational counter-radicalization policies of the selected states. Accordingly, the idea of safe spaces/places for students' learning appears to be rhetoric rather than reality.

Therefore, the findings suggest explicit and wide usage of the concept of human security and its principles in the educational counter-radicalization policies/strategies of the selected cases. This article suggests that some new questions that human security poses for the problem of security would not only be useful in amending the relevant policies; they also have a potential to enhance our understanding of the security-related shortcomings associated with the policies. The new human security questions are: security of whom? security from what? security by what means? and security as perceived by whom?

On the question security of whom, human security would demand that the state and intergovernmental actors make persons rather than states the referent objects of security in handling radicalization issues. The emphasis herein is on personal security – security of the whole human person – including the physical and the psychological. Accordingly, the state and intergovernmental actors need to see the students and/or their parents as the referent objects of security that have bodily and psychological feelings as opposed to the territorial integrity of the states that lack such things (cf. Gasper and Gómez, 2015; Gasper, 2005). Moreover, the rights of the students and/or their parents should be considered from human security perspectives within counter-radicalization policies/strategies. This is necessary, as human security teaches that security in today's world entails “freedom of individuals from the insecurity resulting from [all] human rights violations” (MFA, 2009, 37), including the rights of individual students to safe spaces/places in education. Accordingly, scholars and policymakers need to rethink security relating to educational counter-radicalization policy within the human rights horizon (cf. Adebayo and Mansikka, 2018) more than ever before. Generally, the question of security of whom suggests that holistic security of individual persons could enhance state security. This could in turn result in greater attentiveness to matters of human rights and development (cf. Odutayo, 2016).

The question security from what suggests that security threats go beyond violence against the physical to a host of other threats that could affect the well-being of the human person (Tadjbakhsh and Chenoy, 2007; Adebayo and Mansikka, 2018). It seems many European authorities are yet to give adequate attention to this question in combating terrorism. This is perhaps the reason why the governments have not seen the counter-radicalization policy that potentially promotes fear and indignity as security threats in education spaces. Hence, we now need to emphasize the viewpoint of human security, stating that a feeling of insecurity for many people today “arises more from worries about daily life than from the dread of a cataclysmic world event” (UNDP, 1994, 22). Accordingly, the question security from what needs to be adapted to the context of safe spaces/places in education. The question of safe spaces/places from what in education should give adequate attention to what could psychologically and physically impact the students in terms of security in countering radicalization.

While education could be used to counter negative radicalization (e.g., Ghosh et al., 2016) and enhance human security (e.g., Adebayo and Mansikka, 2018), we need to challenge the educational policing through which the European state/intergovernmental actors seek to prevent terrorism. We must not assume that education is not a gun, so it is not dangerous. The use of education as a means of security could be more dangerous than a bomb blast in the long run if not used according to the principles of human security. Accordingly, this research suggests that the policing-oriented counter-radicalization strategy in education could trigger insecurity and negative security-oriented education for citizenship more strongly than was previously acknowledged in the literature. This leads to the next question – security by what means? As seen above, I have reframed this question for the purpose of this research: security by what type of educational means?

The take in this article is that the use of education in preventing terrorism should not include policing-oriented teaching, as teachers are not security professionals like criminologists or intelligence officers. Education for security is meant to create and promote enabling social, psychological and intellectual conditions for defences, within and among learners, against violent behaviour and ideology (cf. UNESCO, 2017, 22). Such enabling conditions should provide safe spaces/places for intellectual contestations, diverse worldviews and dissenting opinions in schools, including those that might be against what could be regarded as national values (cf. James, 2019; Qurashi, 2018). This is necessary if education is not to drive controversial/sensitive but security-driven and problem-solving conversations underground (cf. Faure-Walker, 2019; Ragazzi, 2017, 41; Jagland as cited in Ragazzi, 2017, 9; CoE and EU, 2015, 13–14). The fact is that diverse good values across countries and continents are inevitable and invaluable in citizenship education that could enhance security. As indicated elsewhere, “good” values and citizenship are not the exclusive preserve of any nation (cf. Adebayo, 2019). Accordingly, a sustainable P/CVE strategy requires every nation to avoid or discard the policy suggesting to its citizens/residents that any person opposing its values is a potential terrorist. Adopting or maintaining such a policy by individual nations could generate mutual suspicion, animosity, inflammatory behaviour and human insecurity among the people across the world. Policymakers and scholars should consider the foregoing in matters of counter-radicalization policies in education.

If security is about the people, then their perceptions of security, not those of state actors, should be central. Hence the question: security as perceived by whom? Putting oneself in the people's perspective, the policing policy in education would easily be seen as a threat to freedom from fear and indignity and the right to a safe learning environment of innocent students. Perceiving security primarily from the perspective of the state/intergovernmental actors seems to have also led to the recent UK and EU definitions of radicalization that neglect the fact that the whole essence of educating people is to inspire (positive) radical thinking (cf. Sukarieh and Tannock, 2015). Accordingly, the question – security as perceived by whom? – is invaluable as one seeks to rejig policing-inclined counter-radicalization policies in education in favour of human security.

Essentially, the policies of the European contexts under investigation need to be rejigged in view of human security in explicit terms. The notion of “explicit” is very significant here, so the concepts in focus could take hold, resonate and clarify the issues of concern (cf. Kaldor et al., 2007). Individual state efforts and a strong synergy between states and intergovernmental actors (e.g., the UK, Finland, the EU, the UNDP, UNESCO, the UN) are necessary to have a common and an effective and enduring front on this matter. Brexit or no Brexit is not an issue here, as the UK is determined to “continue to play a leading international role in countering terrorism during and following the UK's exit from the EU. We will seek to maintain deep and close cooperation with European partners on … security matters” (HM Government, 2018, 30). Correspondingly, the EU, through its RAN, has always been willing to liaise with both its Member States and non-Member States regarding counter-radicalization initiatives (EC n.d.).

## 7 Scope, limitations and future directions of research

Given that the primary data utilized in this research is of macro-level policy documents, its findings do not necessarily reflect the reality at the micro-level.

Although this work uses descriptive discourse analysis, its constructivist assumption and constructivist-oriented notion of human security give its analytic viewpoint a degree of power of language that challenges the elements of the state security approach in the selected documents. It is noteworthy that human security (at its descriptive level) normally challenges state security. Related to this, the consideration for language usually employed in critical or descriptive discourse analysis and the inherent power of language give this work a degree of power in drawing conclusions on how the language in the selected documents shapes reality (cf. Schreier, 2012, 45–47). Hence, challenging the elements of the state security approach in favour of the pupils (and their parents) is not construed as a critical viewpoint in this research. (A typical critical discourse analysis-based study usually uses language and tone to emphasize and criticize the oppressive practices of the powerful against the less powerful (cf. Schreier, 2012); this approach seems to be very apt if one is to examine the subject of this research from the perspective of critical discourse analysis. Moreover, a typical critical discourse analysis-based study needs to and should use a pertinent critical-oriented conceptual or theoretical framework to support and enhance its analysis, e.g., Adebayo, 2019.) Accordingly, this study does not use the vast literature on critical human security studies (e.g., Chandler and Hynek, 2011).

Following the principles of discourse analysis in general (descriptive and critical), the relevant analyses and discussions relating to how language is not used (Schreier, 2012, 47) in the selected documents in this study are not also construed as critical viewpoints. Such analyses and discussions are simply the outcome of my “sceptical reading” of the selected documents, an approach that is typical of discourse analysis in general (cf. Bryman, 2004, 371). Considering the constructivist assumptions (Patton, 2002) guiding discourse analysis in general (Schreier, 2012, 47) and this form of discourse analysis-based study in particular, the discourse and position canvassed, and the conclusions drawn in this article are constructed; hence, they are relative, not absolute (cf. Bryman, 2004).

The discourse relating to safe spaces/places in this study is restricted to the issue of counter-radicalization policies in education. It does not incorporate the general debates about, and theories of, safe spaces as discussed in Flensner and Lippe (2019). Moreover, this paper does not include the debates about securitization and widening of security studies vis-à-vis education-related subject as demonstrated in Adebayo and Mansikka (2018).

This article emphasizes Prevent documents, as it focuses on preventive counter-radicalization initiatives. The UK's Prevent documents receive the most attention in this study, as the UK's Prevent policies are arguably the most influential, elaborate and problematic policing-related policies. Moreover, only the UK educational policing is statutory in Europe and countries like Finland are only beginning to model the UK's policing-related Prevent (Ragazzi, 2017) with significantly less elaborate and problematic documents (see Tables 1, 2, 3 and 4 above).

Given that the findings of this research are based on macro-level policy documents, a further study dealing with collection of data at the micro-level should be carried out. Such a study could provide a better understanding of the subject under investigation. This study only provides a clear “snapshot” (Ruane, 2016; Labaree, 2019) of the policy issue of concern, as its design is cross-sectional. Hence, a further study employing a longitudinal design is needed to have a detailed historical sequence of events concerning the subject under investigation (ibid.; Bryman, 2004). As this work employs a descriptive approach, I recommend a further study using a critical approach. Such a further study could help to better appreciate the need to use human security principles and language in matters of counter-radicalization policies in education.

In addition, the paper recommends a country-specific study employing case study design for a detailed and intensive analysis of the subject matter (Bryman, 2004; Labaree, 2019). Such studies could enhance our understanding of the subject matter vis-à-vis each country. Given that this article reveals that the policing in education in the counter-radicalization policies of the EU, the UK and Finland negates the notion of human security, this study could be a guide to study the situation in other European – EU and non-EU – contexts using similar policing policies in education. As such, this research could be regarded as the beginning of making a case for human security regarding educational counter-radicalization policing policies in Europe and perhaps beyond.

## Declarations

### Author contribution statement

G. O. Adebayo: Conceived and designed the experiments; Performed the experiments; Analyzed and interpreted the data; Contributed reagents, materials, analysis tools or data; Wrote the paper.

### Funding statement

This research was supported by The Finnish Cultural Foundation, Helsinki, Finland; the grant number is 00200149. The Article Publishing Charge was paid by the 10.13039/100007797University of Helsinki, Helsinki, Finland.

### Data availability statement

Data included in article/supplementary material/referenced in article.

### Declaration of interests statement

The authors declare no conflict of interest.

### Additional information

No additional information is available for this paper.

## Footnotes

1 The UK is regarded as an EU Member State in this research as the examined issue and data vis-à-vis the country deal with the pre-Brexit era – before 31 January 2020.

2 Human security is explained in more detail in the conceptual framework section (see below).

3 See below for more about how the UK impacts educational counter-radicalization policy in Europe.

4 See the conceptual framework of this study (below) for more details about the psychological dimension of human security.

5 The EC is the executive branch of the EU.

6 It is understood in this article that Kosovo is only recognized as a sovereign state by a section of the national and intergovernmental authorities of the world (cf. Ragazzi, 2017).

7 Qurashi mostly uses the term “surveillance” (Qurashi, 2018); “surveillance” is central to “policing” (ibid., 3).

10 The remaining (three) strands of the UK CONTEST – Protect, Pursue and Prepare (Home Affairs Committee, 2009) – are not the main focus of this investigation.

11 The other three are Detection, Combating and Incident Management, but these are not the main focus in this paper (MoI, 2018, 19–20).

12 See more about safe spaces in educational counter-radicalization policy in the section below.

## Footnotes

8 Cross-sectional data collection process may last for days or weeks, especially if one works with a large sample (Ruane, 2016, 79) as this study does.
